# Interactions Elicited by the Contradiction Between Figure Direction Discrimination and Figure-Ground Segregation

**DOI:** 10.3389/fpsyg.2018.01681

**Published:** 2018-09-06

**Authors:** Nobuhiko Wagatsuma, Mika Urabe, Ko Sakai

**Affiliations:** ^1^Faculty of Sciences, Toho University, Funabashi, Japan; ^2^Department of Computer Science, University of Tsukuba, Tsukuba, Japan

**Keywords:** figure-ground segregation, border ownership, psychophysical experiment, eye movement, perceptual organization

## Abstract

Figure-ground (FG) segregation that separates an object from the rest of the image is a fundamental problem in vision science. A majority of neurons in monkey V2 showed the selectivity to border ownership (BO) that indicates which side of a contour owns the border. Although BO could be a precursor of FG segregation, the contribution of BO to FG segregation has not been clarified. Because FG segregation is the perception of the global region that belongs to an object, whereas BO determination provides the local direction of figure (DOF) along a contour, a spatial integration of BO might be expected for the generation of FG. To understand the mechanisms underlying the perception of figural regions, we investigated the interaction between the local BO determination and the global FG segregation through the quantitative analysis of the visual perception and the spatiotemporal characteristics of eye movements. We generated a set of novel stimuli in which translucency induces local DOF along the contour and global FG independently so that DOF and FG could be either consistent or contradictory. The perceptual responses showed better performance in DOF discrimination than FG segregation, supporting distinct mechanisms for the DOF discrimination and the FG segregation. We examined whether the contradiction between DOF and FG modulates the eye movement while participants judged DOF and FG. The duration of the first eye fixation was modulated by the contradiction during FG segregation but not DOF discrimination, suggesting a sequential processing from the BO determination to the FG segregation. These results of human perception and eye fixation provide important clues for understanding the visual processing for FG segregation.

## Introduction

Figure-ground (FG) segregation that separates a figural object from background is a fundamental step toward surface construction, shape coding, and object representation (Wagemans et al., 2012). Previous studies have reported that the responses of early- to intermediate-level visual areas with neurons selective to the direction of figure (DOF) were a basis for FG segregation (e.g., Lamme, 1995; Sajda and Finkel, 1995; Zhang and von der Heydt, 2010; Poort et al., 2012). Border ownership (BO) that indicates which side of a contour owns the border might be a precursor of FG segregation. Zhou et al. (2000) demonstrated that a majority of cells in V2 are selective to BO. The determination of BO appears to play an important role for the perceptual discrimination of DOF along a local contour (Wagatsuma et al., 2008; Sakai et al., 2012). The receptive fields of the BO-selective cells in V2 are small as their typical diameter is around 4°. They appear to represent the DOF within a restricted local area, though they appear to integrate surrounding information for the computation of BO (Zhou et al., 2000; Sakai and Nishimura, 2006; Dong et al., 2008; Martin and von der Heydt, 2015; von der Heydt, 2015). A natural hypothesis to bridge local DOF along a contour and global FG might be a spatial integration of BO signals for the construction of a surface that indicates figural region. A recent study has suggested that the population responses of BO-selective cells might underlie the global FG segregation and the neural representation of the figural regions (Nakata and Sakai, 2012; Hasuike et al., 2016). An alternative hypothesis for establishing a global figural region might be a mechanism independent of that for the BO determination. A key to understanding the mechanisms for the segregation of figure from ground lies in investigating the difference in spatiotemporal characteristics between the local DOF discrimination and global FG segregation.

Characteristics of eye fixations and movements are expected to provide insightful suggestions for the investigation of the neural mechanism underlying the perception of local DOF discrimination along a contour and global FG segregation. The location and duration of eye fixations and movements are often utilized as the perceptual indices for understanding a variety of fields (e.g., Chujo et al., 2016; Zimmermann et al., 2018), especially, the strategy of visual processing reflects in the characteristics of eye movements and fixations (e.g., Henderson, 1993; Rayner, 1998; Itti and Koch, 2001; Straube et al., 2010; Sheridan and Reingold, 2017). If perception of DOF is determined based on local processing while that of FG is global, the strategy of eye movements could be different.

In our previous psychophysical study, we investigated the properties of perception and eye movement in local DOF discrimination and global FG segregation for black-and-white natural images (Wagatsuma et al., unpublished). The analyses of responses showed distinct properties in eye movements but similar correctness in perceptual responses. During FG segregation in comparison to DOF discrimination, the spatial range of saccades was significantly greater while the number of saccades was similar, suggesting a strategy that gaze moves rapidly to farther locations for the determination of global FG compared to that of local DOF. Although the results of visual perception and eye movements support distinct mechanisms for FG segregation and DOF discrimination, no clue for their underlying neural mechanisms has been provided.

**FIGURE 1:**
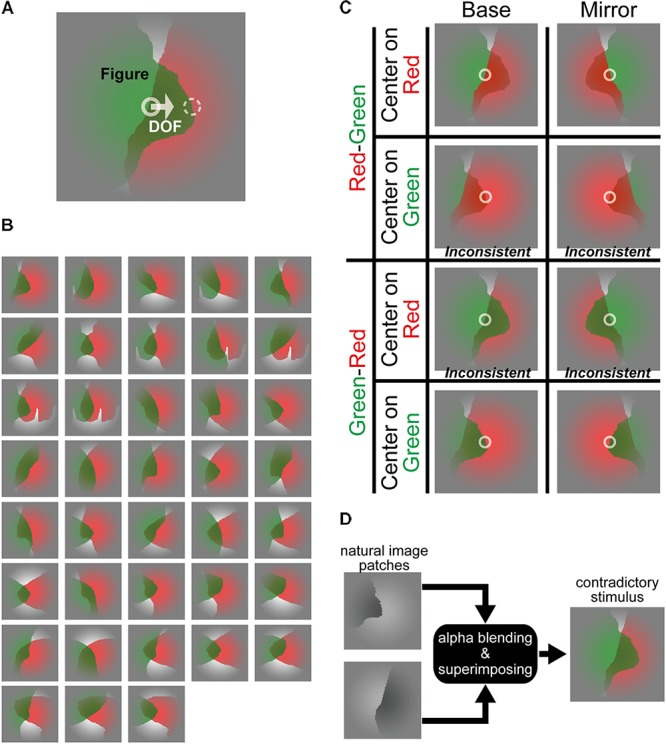
Stimuli in which translucency induces contradictory DOF and FG. **(A)** An example of the contradictory stimulus. In this example, a green, translucent surface was placed physically in front of a red surface, indicating that the *green* surface is perceived as figure. On the other hand, if we focused on the contour at the stimulus center indicated by a white solid circle, the DOF from the contour at the center appears toward right, indicating that the occluded *red* surface owns this border. By contrast, DOF at the contour shown by a white dashed circle and FG were consistent. For generating the consistent stimulus, a contour shown by the white dashed circle fell onto the center of the stimulus by translating horizontally. The solid and dashed circles were drawn on the contours of the back (red) and front (green) surface, respectively. **(B)** Thirty-eight types of the stimuli. These stimuli were generated based on natural image patches (Sakai et al., 2015; Wagatsuma et al., unpublished). **(C)** Four variations of the stimuli: (1) the original stimuli (Base), (2) mirror images with respect to the vertical midline (Mirror), (3) the opposite depth order for the red and green regions (“Red-Green” or “Green-Red”), and (4) horizontal translation (consistent-contradiction, “Center on Red” or “Center on Green”). “Green-Red” and “Red-Green” mean that the green and red regions are physically in front of the other, respectively. “Center on Green” and “Center on Red” mean that the contours of green and red regions are allocated on the center of the stimulus, respectively. White circles represent the center of these stimuli. The first row (Red-Green and Center on Red) is a consistent case where the *red* surface is physically in front of the green, and DOF at the center points toward *red* surface (the right and left for Base and Mirror conditions, respectively) (Figure, DOF = Red, Red). The second from the top (Red-Green and Center on Green) is a contradictory case where the *red* surface is in front, but the DOF at the center points toward the *green* surface (F, D = R, G). The third and fourth rows are contradictory (F, D = G, R) and consistent (F, D = G, G) cases, respectively. These contradictory and consistent cases were defined based on the physical properties of the stimulus. The α value that determines the translucency was then modified for each participant (see section “Materials and Methods” and **Supplementary Material**), if necessary, so that their perceptual depth order of the surfaces agreed with the physical order. Note that the depth order of stimuli in print may appear different from those presented in the display at the laboratory. **(D)** An illustration of the generation of a stimulus for this experiment. Two filled patches with natural shapes were superimposed by alpha blending to generate translucent stimuli.

In order to examine the mechanism for FG segregation through analyses of perceptual responses and eye fixations, we performed psychophysical experiments with novel stimuli in which translucency induces contradictory DOF and FG (**Figure 1**). First, we examined the perception of DOF and FG during a short presentation of the stimulus. We expect distinctly different performance (easiness/difficulty) between DOF discrimination and FG segregation because we hypothesize the contribution of distinct mechanisms for the determination of BO and FG. Second, we analyzed the spatiotemporal characteristics of eye fixation during the presentation of the contradictory stimuli. If the two mechanisms were sequential, the one prior to the other might not be affected by the contradictory signal of the other, while the latter might be modulated by the contradiction. We analyzed the duration of the first fixation (DFF) on figural and ground regions. Our previous study has shown that DFF is an appropriate index for representing the gaze during the task for DOF and FG determination (Wagatsuma et al., unpublished). The DFF is expected to exhibit modulation by the contradiction during FG segregation but not DOF discrimination because BO determination is hypothesized to be determined prior to FG segregation. Our analyses showed that the perceptual performance is better in DOF discrimination, and that DFF during FG segregation is modulated by the contradiction but not DOF discrimination, suggesting a sequential processing from BO to FG. Our analyses of perceptual responses and eye fixations provide psychophysical evidence for clarifying the visual processing in the perception of figure out of ground.

## Materials and Methods

### Participants

We examined psychophysically the interactions between local DOF discrimination and global FG segregation for investigating the mechanisms underlying the perception of figural region. Four male and two female volunteers in their twenties with normal vision participated in the experiments. We asked volunteers whether they have been diagnosed as color blind, including the result of the color vision test that was performed as a part of the health examination in their elementary schools. None were diagnosed as color blind or partial color blind. The volunteer who did not show meaningful results in the color adjustment for transparency (see **Supplementary Material**) did not participate in the experiments, which assured that all participants perceived the correct depth order from the color transparency used in the experiments. The participants were familiar with visual psychophysics but not aware of the purpose of the experiments. The experiments were approved by the Research Ethics Committee of the Faculty of Engineering, Information and Systems, University of Tsukuba, in accordance with the Code of Ethics of the World Medical Association (Declaration of Helsinki). Prior to the experiments, all participants gave written informed consent as approved by the Research Ethics Committee of Faculty of Engineering, Information and Systems, University of Tsukuba.

### Apparatus

The stimuli were presented on a 24.1″ LC monitor (ColorEdge CG242W; EIZO Corporation) at a refresh rate of 60 Hz. The monitor was placed at a distance of 60 cm in front of the participants. Eye movements were monitored *via* an eyetracker machine (Tobii Eye Tracker X60; Tobii Technology AB) at a nominal sampling rate of 60 Hz. In preliminary tests, the eyetracker machine recorded 51.6 data per second on average. These experimental systems and settings were identical to those in our previous psychophysical study (Wagatsuma et al., unpublished).

### Stimulus

We investigated psychophysically the characteristics of perception and eye movement in DOF discrimination and FG segregation with stimuli consisting of natural shapes in which DOF and FG are contradictory. In this study, DOF discrimination is defined as the determination of the figure direction with respect to a local contour. FG segregation is defined as the determination of a figural region within a visual field. We generated novel stimuli in which DOF and FG are contradictory. The stimuli consist of two overlapping natural shapes with a translucent surface in front, as an example is shown in **Figure 1A**. In this case, a green, translucent surface is physically placed in front of a red surface, so that the green surface is perceived as figure (FG segregation = green). Now, the participants are asked to judge the DOF at the stimulus center (indicated by a small circle with a white solid line; not shown in the experiment). Because this edge is the contour of the red surface which is occluded by the green surface, the perceived DOF of the local contour at the center is right toward the occluded red surface (BO discrimination = right = red). Therefore, the DOF and FG are contradictory. Note that the determination of the DOF along the local contour is independent of the depth order of two translucent surfaces. Other example stimuli are shown in **Figures 1B,C** and the details of stimulus generation are described below.

The stimuli with a translucent surface were generated based on the combination of two natural image patches (Sakai et al., 2015; Wagatsuma et al., unpublished). We selected arbitrarily two image patches from the Berkeley Segmentation Dataset (Martin et al., 2001; Fowlkes et al., 2007), with the exclusion of those including a whole object or characteristic parts of an object in which participants tell instantaneously a figural region from their knowledge (Sakai et al., 2015). We filled the figure region of one patch with either green or red, and that of the second patch with the other color. We confirmed with a preliminary experiment that all participants showed nearly perfect correct discrimination for the filled patches. We then superimposed the two filled patches by alpha blending (Porter and Duff, 1984) to generate translucency, as illustrated in **Figure 1D**. **Figure 1B** illustrates a set of these stimuli.

The *consistent* and *inconsistent* stimuli were generated by the horizontal translation of the patches. A patch was placed so that a contour of the region given by a mixture of red and green passed through the center of the stimulus (indicated by an imaginary white circle in **Figures 1A,C**). Note that the participants judge the DOF at the stimulus center, thus whether consistent or inconsistent depends on what is presented at the stimulus center. If a patch (say, red) is placed in front of the other and its contour passes through the stimulus center, the DOF along the local contour at the center and FG are consistent (a consistent stimulus), as shown in the top row of **Figure 1C**. To generate inconsistency between DOF and FG, we translated horizontally the stimulus so that a contour of the other patch (green) fell onto the center (an inconsistent stimulus), as shown in the second row of **Figure 1C**. In the case of **Figure 1A**, the DOF from the contour at the center (toward the occluded red region) is inconsistent with FG (green) because the surface in back (red) is placed so that its contour passed through the center. If we move the patch to the left so that the location indicated by a dashed white circle fall onto the stimulus center, the DOF at the center (front green region) is now consistent with FG (green). Whether a surface appears in front or back depends not only on the physical property but also on the perception of individual participants (Beck et al., 1984). Therefore, we modified permeation rates for each participant to assure that the perceptual depth order of surfaces was identical to the physical property for all participants (see **Supplementary Material**).

A superimposed patch consists of four regions with distinct colors as shown in **Figure 1**: red and green representing distinct figures, a mixture of red and green due to the translucency of a surface in front, and white representing ground. The generation of translucency by the superimposition of the two filled patches based on alpha blending (Porter and Duff, 1984) was given by following processing (see **Figure 1D**):

(1) $$I_{\mathrm{back}}^{\prime }\;=\;\alpha_{\mathrm{back}}I_{\mathrm{back}}\;+\;(1-\alpha_{\mathrm{back}})W$$

(2) $$S_{\mathrm{superimpose}}\;=\;\alpha_{\mathrm{front}}I_{\mathrm{front}}+(1-\alpha_{\mathrm{front}})I_{\mathrm{back}}^{\prime }$$

(3) $$S_{\mathrm{front}}\;=\;\alpha_{\mathrm{front}}I_{\mathrm{front}}\;+\;(1-\alpha_{\mathrm{front}})W$$

(4) $$S_{\mathrm{back}}\;=\;\alpha_{\mathrm{back}}I_{\mathrm{back}}\;+\;(1-\alpha_{\mathrm{back}})W$$

where *I*_front_ and *I*_back_ represent the intensity of figure regions of the original natural image patches in front and back, respectively. α_front_ and α*_i_* indicate the permeation rates for *I*_front_ and *I*_back_, respectively. *W* indicates the intensity of the ground region of the patches filled with white. *S*_superimpose_ represents the intensity of the overlapping region when *I*_front_ is superimposed on *I*_back_. *S*_front_ and *S*_back_ show the intensity of the translucent images with permeation rates α_front_ and α_back_ for *I*_front_ and *I*_back_, respectively. Because the perception of transparency depends on individuals (Beck et al., 1984), the α values for each participant were assigned to generate the most transparent surfaces based on the results of the preliminary experiments specifically designed for this purpose (see **Supplementary Material**).

We generated 38 stimuli with distinct combinations of shapes as shown in **Figure 1B**. To cancel out possible biases in the perception, we prepared the mirror images with respect to the vertical midline, and the images with the opposite depth order for red and green regions, as illustrated in **Figure 1C**. As described previously, we also generated images with horizontal translation in order to generate inconsistency between DOF and FG. We prepared 304 test stimuli in total (38 types of shape × 2 mirror images × 2 color patterns × 2 patterns for the translation).

### Experimental Procedure

**FIGURE 2:**
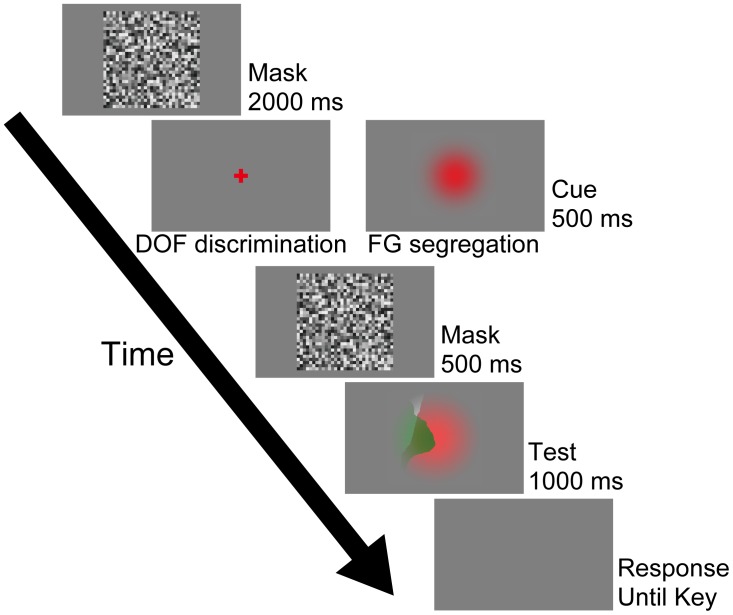
Procedure used in the psychophysical experiment. In this work, this procedure program was run after the calibrations. Participants were instructed to judge the DOF along the local contour passing through the screen center during the DOF discrimination task, which was instructed by the presentation of the red cross cue (left panel of the Cue). By contrast, the red Gaussian cue was given for the FG segregation task (right panel of the Cue). Under this condition, participants were asked to report which region, the red or green, appeared in front of the other. These cues were not represented at the location of the fixation point. Participants were instructed to respond to these tasks at the end of each trial (Response screen) using a two-alternative forced choice (2AFC) paradigm. Except for the presented stimuli in Test screen, this procedure is identical to that of our previous study (Wagatsuma et al., unpublished). See text for details.

In this work, DOF discrimination is defined as the determination of the figure direction along a local contour at the screen center (Wagatsuma et al., 2008, 2013; Sakai et al., 2012), the task of which needs no more than the inspection of a local area around the stimulus center. By contrast, FG segregation is defined as the global perception of a figure, the task of which needs the inspection of the entire field of the stimulus. There were two types of tasks with distinct instructions for the participants to answer DOF or FG, as details described later. **Figure 2** shows the procedure of our psychophysical experiments. We recorded both perceptual responses and eye movements during the presentation of the stimuli (Test). The experiments started with the presentation of a mask display at the center of the screen for 2000 ms (Mask). After the disappearance of the mask, in order to instruct the type of task, a cue stimulus (a cross for DOF and a Gaussian circle for FG) appeared at the center of the screen for 500 ms (Cue). Note that we did not ask the participants to fixate the cross or circle in the cue stimulus because our aim includes the examination of the spatiotemporal characteristics of eye fixations during the DOF discrimination and FG segregation. Subsequently, another mask was presented for 500 ms and a stimulus (9° × 9°) was presented at the center for 1000 ms (Test). At the end of each trial, a blank screen was presented until the detection of the participants’ perceptual responses. Except for the stimuli in Test screen, this procedure is identical to that of our previous study (Wagatsuma et al., unpublished). This procedure was controlled by MATLAB through Psychotoolbox (Brainard, 1997; Pelli, 1997).

The participants were instructed to notice whether a trial requires DOF discrimination or FG segregation from the cue stimuli (the cross and circle, respectively), and then perform the trial accordingly. The DOF discrimination task was instructed by the presentation of a red cross (2° × 2°: left panel of the Cue on **Figure 2**) at the center of the screen. In this task, the participants were asked to judge the local DOF (right or left) along the contour passing through the center of the screen. They were asked to press either the right or left arrow key with their right hand when the perception of the local DOF along the contour at the stimulus center was to the right or left, respectively. The FG segregation task was instructed by a red Gaussian circle (right panel of the Cue on **Figure 2**). In this task, the participants were asked to report which region, the red or green, appeared in front of the other. They were asked to press either the ‘C’ or ‘Z’ key with their left hand when the frontal surface was red or green, respectively. In our preliminary experiment, we tested whether the perceptual responses and eye movements for the FG segregation task depended on the dimension of the cue. Although the larger cue was easier for some participants to distinguish, our preliminary experiment did not show a dependence of perceptual responses and eye movements on the dimension of the cue (9° × 9° vs. 2° × 2°). In the following sections, we describe the results obtained with the 9° × 9° cue. Note that we did not instruct the participants to fixate these cues. However, the results indicated that the participants tended to gaze at the region around the center of the screen where the cue was presented at the onset of the Test stimulus. We gave participants a five minute break between each session lasting about ten minutes. The experiment was limited to 40 consecutive minutes per day and repeated up to five days when necessary.

A total of 304 stimuli were presented in the experiment. The combination of two types of cue (DOF discrimination and FG segregation tasks) and three repeats yielded a total of 1824 trials. The order of the presentation of the condition (8) and shapes (38) of stimulus, and instruction (cue) were randomized. We excluded the trials from the analyses that failed to provide enough points of eye position during the presentation of stimulus (*n* > 10). Prior to each experimental session, the eyetracker machine was calibrated to assure the accurate recording of the spatiotemporal characteristics of eye fixations. We reran the calibration until the machine successfully detected all 9 points on a grid within the location of stimulus presentation, unless the participant gave up the calibration and quitted the experiment. The overall accuracy (SD) of the gaze location was 0.74°.

## Results

In order to investigate the neural mechanisms underlying the perception of DOF and FG, we performed psychophysical experiments with novel stimuli in which translucency induces contradictory DOF and FG. We examined the correct perception rate of DOF and FG, and the spatiotemporal characteristics of eye fixations, specifically, the DFF on figural and ground regions. Because BO is considered to be determined prior to FG segregation, we expect that DOF discrimination exhibits a higher correct rate than FG segregation under a short presentation of stimuli. More importantly, we also expect that the contradiction modulates eye movements in FG segregation but not in DOF discrimination.

### The Correct Rate in the Perception of DOF Discrimination and FG Segregation

We compared the rates of correct responses in DOF discrimination and FG segregation in order to examine whether the easiness of the perception in a short period of presentation differs between the two. If we observed a marked difference in the correct rate between DOF discrimination and FG segregation, we would expect different processing for the determination of local DOF and global FG. Together with the analysis of eye fixation, the analysis of perceptual performance is expected to provide insights into the neural mechanisms underlying DOF discrimination and FG segregation.

Under the DOF discrimination task, the participants judged the local DOF (right or left) along the contour passing through the center of the screen. If the perceived DOF was toward the surface whose contour passed through the screen center (a white arrow in **Figure 1A**), we considered this response correct. Under the FG segregation task, participants reported the color of the surface that appeared in front of the other (“Figure” label in **Figure 1A**). If the surface physically located in front was perceived as figure, we considered this response correct. Because of the transparency of a surface, DOF and FG could be physically contradictory or consistent in the stimuli.

**FIGURE 3:**
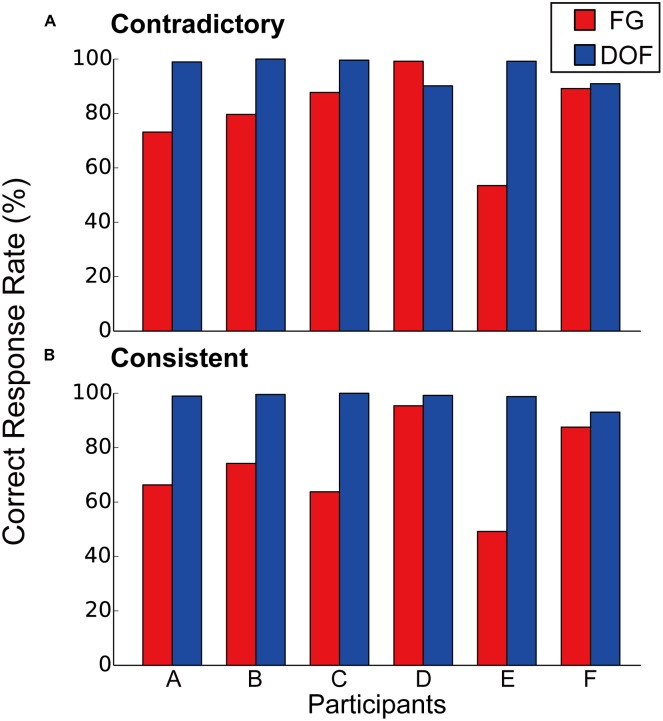
Correct rates of contradictory **(A)** and consistent **(B)** stimuli for FG segregation (red bars) and DOF discrimination (blue bars). For the contradictory stimuli **(A)**, five participants (A, B, C, E, and F) indicated greater correct rates in DOF discrimination compared to those in FG segregation. The perceptual responses to the consistent stimuli **(B)** were similar to those to the contradictory stimuli.

The measured correct rates for FG segregation and DOF discrimination are shown in **Figure 3**. Five participants (A, B, C, D, and F) showed the correct responses over the chance rate (50%) in both DOF discrimination and FG segregation. **Figure 3A** shows the correct rates for the contradictory stimuli. We observe that the correct rates in DOF discrimination were greater than those in FG segregation for five participants (A, B, C, E, and F). The mean correct rate among participants was significantly greater in DOF discrimination than FG segregation (pairwise *t*-test, *t*(5) = 2.05, *P* = 0.048). **Figure 3B** shows the results for the consistent stimuli. The statistical analysis for the consistent stimuli showed the result similar to the contradictory stimuli (pairwise *t*-test, *t*(5) = 3.48, *P* < 0.01). A two-way ANOVA with factors of tasks (DOF discrimination and FG segregation) and conditions (contradictory and consistent stimuli) showed significance in the tasks [*F*(1,20) = 18.47, *P* < 0.01] but not in the conditions [*F*(1,20) = 0.37, *P* = 0.55]. Further analyses with a factor of the condition for each task showed no significance [*F*(1,22) = 0.21, *P* = 0.65]. These results indicate that the contradiction between DOF and FG does not account for the difficulty in the FG task. These characteristics of human perception indicate that the perception of local DOF was predominant over that of global FG, suggesting different mechanisms for the determination of BO and FG.

### The Duration of the First Eye Fixation on Figural and Ground Regions

To investigate the effects of interactions between DOF discrimination and FG segregation evoked by the contradiction between the two, we examined the characteristics of eye movements and fixations during stimulus presentation. Specifically, we analyzed the DFF to figure and ground regions in DOF discrimination and FG segregation. Our previous psychophysical study reported the longer DFF in a figural region than a ground region (Wagatsuma et al., unpublished). We expect to observe the modulation of such tendency evoked by the contradiction between FG and DOF because the contradiction may confuse one (FG or DOF) and evoke unusual eye fixations if it depends on the other. For instance, if the determination of BO precedes processing for FG organization, the contradiction evokes the modulation of DFF in FG segregation but not DOF discrimination. The results in the previous section showed no difference in the correct rate between the consistent and contradictory condition, indicating similar overall difficulty between the two conditions. The two conditions shared the identical task within each of DOF discrimination and FG segregation. Therefore, the difference between the consistent and contradictory conditions is independent of task and the difficulty inherent in the conditions.

**FIGURE 4:**
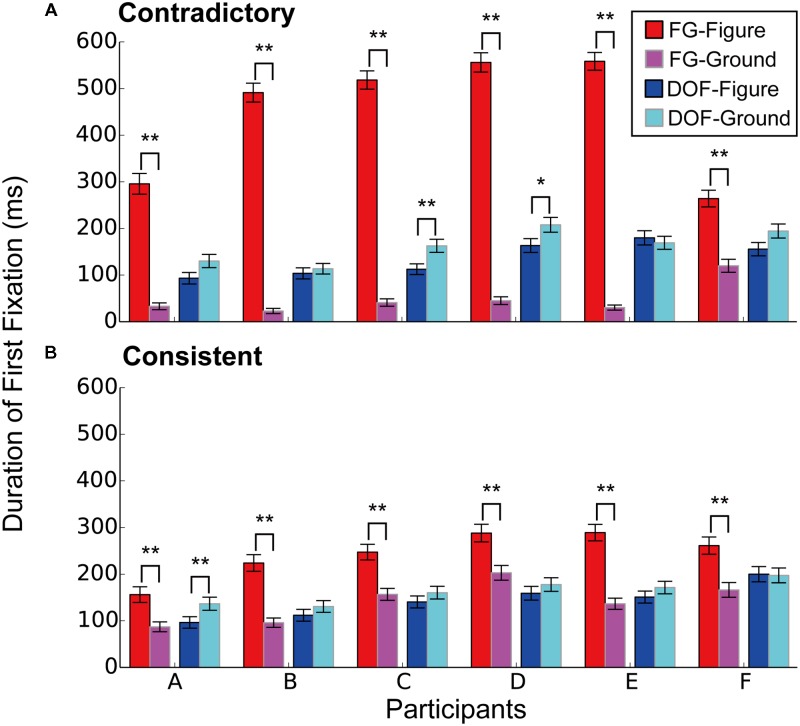
The duration of first fixation (DFF) to the figural and ground regions for the DOF discrimination and FG segregation. **(A)** The DFF in the contradictory condition. **(B)** The DFF in the consistent condition. Red bars indicated the mean DFF to the figural region under the FG segregation task. Blue showed DFF to the figural region under the DOF discrimination task. Magenta represented DFF to the ground under the FG task. Cyan meant DFF to ground under DOF task. Asterisks indicate significant differences between two regions (*t*-test: ^∗∗^*P* < 0.01; ^∗^*P* < 0.05).

We defined saccade according to Engbert and Kliegl (2003) in which eye movements over a greater distance were considered as saccade (the threshold λ = 6 in Engbert and Kliegl, 2003). We monitored eye movements through the eyetracker machine at the sampling rate of 60 Hz, which was slightly sparse for the examination of microsaccades. Given the sampling rate, we consider that our experimental data reflect saccades but not microsaccades. Therefore, in this study, we analyzed only the large shift of eye fixation such as the DFF. The successive small movements between the saccades were considered as a single fixation. We presented stimuli within a 9° × 9° region at the screen center (see section “Materials and Methods”). This large dimension of the stimulus induced the large shift of eye fixations for exploring the presented stimulus. The DFF to a figural region was defined as the duration that fixation stayed on the perceptual figure-region for the first time after the stimulus onset, and the DFF to a ground region was defined likewise. The measured DFFs to figural and ground regions for FG segregation and DOF discrimination are summarized in **Figure 4**. In the present experiment, half of the stimuli were contradictory between FG and DOF (contradictory stimuli), whereas the other half induce global FG in agreement with local DOF (consistent stimuli) (**Figure 1C**). In the following sections, we analyze the characteristics of DFF in the contradictory and consistent conditions, and compare the results to clarify the modulation by the contradiction. The DFF was categorized by three factors: participants, tasks (FG and DOF) and fixation region (figure and ground), in addition to the condition (consistent and contradictory stimuli).

**Figure 4A** shows the DFF in the contradictory condition. Under the FG segregation task, all participants showed significantly longer DFF in figure regions than that in ground (red and magenta bars in **Figure 4**; *t*-test, A: *t*(568) = 11.2, *P* < 0.01; B: *t*(814) = 22.2, *P* < 0.01; C: *t*(900) = 22.6, *P* < 0.01; D: *t*(868) = 22.9, *P* < 0.01; E: *t*(850) = 26.4, *P* < 0.01; F: *t*(778) = 6.34, *P* < 0.01). These results indicate that, during FG segregation, participants gazed figure regions longer than ground regions. By contrast, under the DOF discrimination task, five out of six participants showed longer DFF in ground regions compared to that in figure (blue and cyan bars in **Figure 4**). However, their differences reached significance only in two participants (*t*-tests, A: *t*(626) = 2.00, *P* = 0.051; B: *t*(760) = 0.60, *P* = 0.549; C: *t*(886) = 2.76, *P* < 0.01; D: *t*(870) = 2.05, *P* < 0.05; E: *t*(866) = 0.515, *P* = 0.608; F: *t*(814) = 1.87, *P* = 0.062). This result suggests that, during DOF discrimination, participants gazed equally at figure and ground regions. These results suggest different strategies and mechanisms for the determination of DOF and FG. **Figure 4B** shows the DFF in the consistent condition. Statistical analyses for the consistent condition showed the results similar to the contradictory condition, as illustrated in the figure.

**FIGURE 5:**
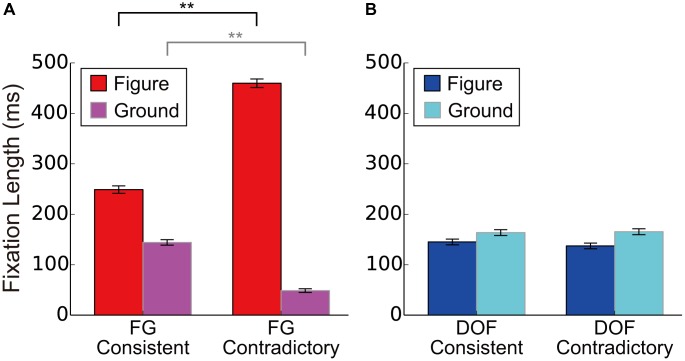
The DFF for the consistent and contradictory stimuli in FG segregation and DOF discrimination. **(A)** The DFF for consistent and contradictory stimuli during FG segregation. We showed mean DFF for six participants. Red and magenta bars meant the DFF to the figure and to ground regions, respectively. **(B)** The mean DFF for consistent and contradictory stimuli during DOF discrimination. Blue and cyan bars meant the DFF to the figure and to ground regions, respectively. Asterisks indicate significant differences between two conditions (*t*-test: ^∗∗^*P* < 0.01; ^∗^*P* < 0.05). Error bars were the standard error of all participants.

To investigate the effects of the contradiction between DOF and FG, we analyzed the dependence of DFF on the contradiction. The DFF for the figural region was longer than that for the ground region in the FG task. This tendency appears greater in the contradictory condition compared to the consistent condition, as observed in **Figure 4**. We summarized the measured DFF for the consistent and contradictory stimuli in FG segregation and DOF discrimination in **Figures 5A,B**, respectively. With the FG segregation task, three-way ANOVA with factors of the condition (contradictory and consistent stimuli), fixation region (figure and ground) and participant showed significance in all main factors and the interaction between the condition and fixation region (*P* < 0.01; **Table 1**). By contrast, the DOF task did not show significance in the condition and the interactions (**Table 2**). These results indicate the significant modulation in the FG task, but not in the DOF task.

**Table 1:** The result of a three-way ANOVA for the FG segregation task with factors of conditions (contradictory and consistent stimuli), fixation regions (figure/ground locations), and participants for the examination of the DFF.

Factor	*df*	Sum of squares	Mean square	*F* value	*P*-value	Partial eta squared
Condition (C)	1	7.83 × 10^6^	7.83 × 10^6^	80.11	<0.001	0.00829
Fixation region (F)	1	1.59 × 10^8^	1.59 × 10^8^	1627.63	<0.001	0.145
Participant (P)	5	1.52 × 10^7^	3.03 × 10^6^	31.00	<0.001	0.0159
C × F	1	5.60 × 10^7^	5.60 × 10^7^	572.84	<0.001	0.0564
C × P	5	3.57 × 10^6^	7.13 × 10^5^	7.29	<0.001	0.00379
F × P	5	1.48 × 10^7^	2.95 × 10^6^	30.16	<0.001	0.0155
C × F × P	5	1.03 × 10^7^	2.06 × 10^6^	21.04	<0.001	0.0110
Error	9588	9.38 × 10^8^	9.78 × 10^4^			


**Table 2:** The result of a three-way ANOVA for the DOF discrimination task with factors of conditions (contradictory and consistent stimuli), fixation regions (figure/ground locations), and participants for the examination of the DFF.

Factor	*df*	Sum of squares	Mean square	*F* value	*P*-value	Partial eta squared
Condition (C)	1	2.67 × 10^4^	2.67 × 10^4^	0.335	0.563	<0.001
Fixation region (F)	1	1.32 × 10^6^	1.32 × 10^6^	16.62	<0.001	0.00141
Participant (P)	5	7.48 × 10^6^	1.50 × 10^6^	18.79	<0.001	0.00791
C × F	1	5.84 × 10^4^	5.84 × 10^4^	0.734	0.392	<0.001
C × P	5	5.47 × 10^5^	1.09 × 10^5^	1.38	0.230	<0.001
F × P	5	3.49 × 10^5^	6.98 × 10^4^	0.877	0.496	<0.001
C × F × P	5	4.05 × 10^5^	8.10 × 10^4^	1.02	0.406	<0.001
Error	9684	7.71 × 10^8^	7.96 × 10^4^			


To further analyze the effects of the contradiction, we analyzed the modulation in detail. With the FG segregation task, we observed significantly longer DFF to the figure region for the contradictory stimuli than that for consistent stimuli [red bars in **Figure 5A**; *t*-test, *t*(4804) = 18.71, *P* < 0.01]. The DFF to the ground region for the contradictory stimuli were significantly shorter than that for consistent stimuli [magenta bars in **Figure 5A**; *t*-test, *t*(4804) = 14.60, *P* < 0.01]. By contrast, with the DOF discrimination task, we observed no significant difference in DFF between the contradictory and consistent stimuli [blue and cyan bars for figure and ground regions, respectively, in **Figure 5B**; *t*-tests; *t*(4852) = 0.99, *P* = 0.322 and *t*(4852) = 0.21, *P* = 0.833, respectively]. The DFF in FG segregation was modulated by the contradiction with DOF, suggesting that the perception of global FG is markedly modulated by the determination of local BO. On the other hand, the DFF in DOF discrimination was not modulated by the contradiction with FG, suggesting that the perception of local DOF is independent of that of global FG. These results appear to be in agreement with our idea of the subsequent neural mechanism where the integration of BO underlies the perception of FG segregation. The possibility of this mechanism will be discussed further in the Discussion section.

## Discussion

We investigated the mechanism underlying the perception of local DOF discrimination and global FG segregation through psychophysical experiments with novel stimuli in which translucency induces contradictory DOF and FG. We analyzed the correct perception rate of DOF and FG, and spatiotemporal characteristics of eye movements. The analyses of the correct rates indicated that the perception of local DOF was dominant over that of global FG. The analyses of eye movement indicated that the duration of first fixation (DFF) in FG segregation was modulated by the contradiction whereas the DFF in DOF discrimination was not. These results provided important insights for clarifying the visual processing in the perception of figure out of ground. Specifically, the results support the notion of an existence of a subsequent neural mechanism where the integration of BO underlies the perception of FG segregation.

Physiological studies have reported that the responses of neurons in V2 and V4 underlie the determination of BO (Zhou et al., 2000; Dong et al., 2008; O’Herron and von der Heydt, 2009; von der Heydt, 2015). Recent electrophysiological recording during texture segregation have reported that activation of V1 neurons enhanced immediately the responses of V4 to figure regions whereas the feedback from V4 modulated that of V1 in a late time window (Klink et al., 2017). Two-photon calcium imaging and electrophysiology of rodents have reported BO modulation in early visual areas and FG modulation in higher cortical areas (Luongo et al., 2017). Computational studies have suggested the roles of the population responses of BO-selective cells for the representation of the global FG segregation (Nakata and Sakai, 2012; Sakai et al., 2012; Hasuike et al., 2016). Such reports led us to hypothesize that the spatial integration of BO signals underlies the organization of global FG representation. Analyses of the present psychophysical experiment exhibited asymmetric modulation for DFF: the contradiction between DOF and FG modulated DFF in FG segregation (**Figure 5A**) but not in DOF discrimination (**Figure 5B**). This result suggests that the establishment of figure undergoes more complex processing in the contradictory condition because the DOF along the contours of the back surface that go behind the front surface could prevent a formation of the front surface. In the consistent condition, the DOF along the contours of the front surface could form the front surface without difficulty. One might argue that the asymmetric modulation of DFF is consistent with the idea that the determination of DOF takes more time with an occluded surface than occluding surface (Sekuler et al., 2000). A psychophysical study reported the dependence of the reaction time on the extent of occluded surface (Shore and Enns, 1997). However, in our stimuli, the surface areas of the occluding and occluded surfaces are identical between the contradictory and consistent conditions. Furthermore, the modulation of DFF has no direct relation to the reaction time. The dependence of the reaction time on occlusion cannot account for the asymmetric modulation of DFF. One might also argue that the difference between the consistent and contradictory conditions could be affected by the difficulty of the tasks for the two conditions. However, the tasks for the two conditions were identical within each of DOF discrimination and FG segregation (**Figure 3**). Therefore, the difference between the two conditions are independent of the difficulty and instruction of task. During DOF discrimination, DFF to ground regions was slightly longer than that to figure independent of the contradiction between DOF and FG (blue and cyan bars in **Figure 4**; the difference did not reach significance). By contrast, during the FG segregation task, we observed significantly longer DFF to figure regions than to ground (red and magenta bars in **Figure 4**). The processing of DOF discrimination might not need the information of figure and ground. Sequential processing from BO determination to FG segregation provides a possible explanation for the characteristics of the DFF.

Physiological experiments have clarified a variety of characteristics of the responses of BO-selective cells. Qiu et al. (2007) demonstrated interactions between selective attention and BO selectivity in the neuronal responses in V2. The grouping structure of presented stimuli modulated temporal characteristics of responses on BO-selective cells (Dong et al., 2008). Martin and von der Heydt (2015) found that selective attention increased the rates of BO-selective cells and decreased the spike synchrony between them. These results implied that selective attention and feedback projections directly influence the activities of BO-selective cells in intermediate-level visual areas. In addition, other physiological studies showed that significant BO signals were induced within 70 ms after stimulus onset (Zhang and von der Heydt, 2010; Williford and von der Heydt, 2016). Grouping-cell models have been proposed to account for these characteristics of BO-selective cells (Craft et al., 2007; Mihalas et al., 2011). The hypothetical grouping cells not only integrate the signals from BO-selective cells but also mediate the feedback projections and selective attention to BO-selective cells. The behaviors of the grouping-cell model agree with those of BO-selective cells (Craft et al., 2007; Zhang and von der Heydt, 2010; Wagatsuma et al., 2016). These physiological and computational works support the important roles of the feedback projections to V2 in the determination of local DOF. However, the grouping cells that mediate feedback signals to BO-selective cells are hypothetical and have not been reported physiologically. Furthermore, these grouping-cell models have not been applied to and tested by complex stimuli such as natural images.

By contrast to the grouping-cell hypothesis, other computational studies have suggested that the surrounding suppression/facilitation observed in early vision (Jones et al., 2001, 2002; Ozeki et al., 2009) underlies the determination of the responses of BO-selective cells (Sakai and Nishimura, 2006; Sakai et al., 2012). In their models, the surrounding organization was crucial in the integration of the luminance contrast that led to the DOF determination with respect to the border. Their model showed the applicability to natural images and reported 67% overall correct determination of BO which was similar to that reported electrophysiologically for the same set of natural stimuli (Williford and von der Heydt, 2016). Sakai and Michii (2013) demonstrated that these surround modulation models with plausible anatomical constraints including feedback reproduced the latency characteristics observed in BO-selective cells (Zhang and von der Heydt, 2010). Their results suggest that multiple factors, such as feedforward signals from V1, feedback from V4 and their interactions, play important roles for the response dynamics of BO-selective cells. It is important to discover the neural mechanisms underlying BO-selective cells and their roles in FG discrimination.

We observed distinct characteristics in eye movements depending on the contradiction between DOF and FG. The results of the consistent condition in the present study showed similar characteristics to our previous study (Wagatsuma et al., unpublished). This is natural because the stimuli presented in the previous study were black-and-white natural image patches that always induced consistent perception between local DOF discrimination and global FG segregation. The stimuli used in the present study induced contradictory DOF and FG at the center of stimuli due to the translucency (**Figure 1**). Such contradictory stimuli are expected to induce interaction between local DOF processing and global FG processing, which would provide useful evidence for understanding the neural mechanisms. The characteristics of eye movements associated with the contradiction are expected to help with understanding the interaction between BO determination and FG segregation. Recent studies have reported that the feedback projections underlie the neural mechanisms of BO determination and FG segregation (Craft et al., 2007; Mihalas et al., 2011; Self et al., 2013; Martin and von der Heydt, 2015; Wagatsuma et al., 2016). Such feedback signals from higher levels of the visual cortex may mediate the structure of objects and the influence of selective attention, which play a fundamental role in the perception of shapes and objects. However, in our psychophysical results, DFF for the figural region under the contradictory condition was significantly longer compared to that under the consistent (**Figure 5A**). This result suggests longer processing time or more complex processing for establishing the figure perception under the contradictory condition, which seems to arise from sequential processing that the integration of BO requires for the organization of FG. The contradictory stimuli appeared to require more complex interactions between feedforwad and feedback signals compared to the conventional stimuli without contradiction. Further computational studies for modulation with the contradictory stimuli are expected to advance our understanding of the neural mechanisms underlying the perception of DOF and FG.

Electrophysiological recordings (Lamme, 1995; Poort et al., 2012) during texture segregation indicated that neurons in V1 and V4 respond more vigorously to figure regions in stimuli than to ground. Interestingly, Poort et al. (2016) reported that the latency of the figure enhancement in V4 (55–85 ms) was markedly earlier than that in V1 (65–100 ms), suggesting the feedback projections from V4 to V1 for the FG modulation. They also reported early enhancement in the representation of object borders in V1 (about 60 ms; Poort et al., 2012). von der Heydt and his colleagues have reported that the BO signals in V2 emerge 60–70 ms after stimulus onset (Zhang and von der Heydt, 2010; Williford and von der Heydt, 2016). These physiological data implied distinct mechanisms for FG segregation and BO determination. However, since the stimuli, procedures, and tasks were different between the two groups, there might be a possibility that the early enhancement in V1 reflected the feedback from BO-selective cells in V2. Further studies on dynamics in early and mid-level visual areas are required to clarify the cortical mechanisms and interactions for BO determination and FG segregation.

Monkey electrophysiology has played critical roles in understanding the mechanisms underlying the neural coding of BO and object (Zhou et al., 2000; Qiu et al., 2007; Zhang and von der Heydt, 2010; Martin and von der Heydt, 2015; Williford and von der Heydt, 2016). Human psychophysics have also provided important evidence for the perception of FG in relation to BO. The observation of BO-dependent tilt aftereffects have suggested that FG is represented by BO-selective cells at early stages in the human visual cortex (von der Heydt et al., 2005; Sugihara et al., 2007; Sugihara et al., 2008). The computational models that aim to understand the neural mechanisms of BO-selective cells have often compared their overall performance with psychophysical measures (Wagatsuma et al., 2008, 2013; Sakai et al., 2012). Visual perception measured by psychophysical experiments seemed to provide important insights for understanding the neural mechanism of BO-selective cells.

Shore and Enns (1997) have reported that highly occluded objects required a longer time for completion than less occluded objects. Interestingly, our previous psychophysical work indicated the irrelevance of the object size to attentional modulation in the perception of DOF (Wagatsuma et al., 2013). It would be interesting to investigate further the neural mechanisms underlying the reaction time for the images with occlusion. In the present study, we measured DFF, not a reaction time, for the stimuli consisting of two overlapping natural shapes with a translucent surface in front (**Figure 1**). Because the consistent and contradictory conditions differed only in the horizontal translation, the spatial extent of the overlapping (occluded and occluding) regions were identical between the two. Therefore, our result cannot be compared with that of Shore and Enns (1997). The present study is, to our knowledge, the first work to report whether the duration of eye fixation to figure or ground region depend on the occluding or occluded condition and their interactions. The modulation of DFF in FG segregation arises from the contradiction between local DOF and global FG rather than the spatial extent of the occluded region.

Our stimuli consisted of four regions with distinct colors (see section “Materials and Methods”). The visual system could rely on color in the FG tasks in addition to the difference in luminance. It is of great interest to clarify the role of color vision in the perception of FG. A reason that we used color rather than gray is its saliency in the perception of transparency. The addition of a blended color improved the impression of transparency, though what blend evoked the perception of transparency depended heavily on individual. Because transparency is a crucial cue for depth order, color vision is considered to play a role also in the determination of FG. It is also possible that colored stimuli could influence eye movements during our tasks. Computational studies have reported that color is one of the visual features that determine the attended location and attentional selection (Itti et al., 1998; Itti and Koch, 2001; Russell et al., 2014), which suggest that the eye fixations could be modulated by the distribution of colors in the present stimuli. Our psychophysical and computational studies suggested also that feature-based attention significantly modulates the perception of DOF (Wagatsuma et al., 2013). If the cues (Cue screen in **Figure 2**) drew feature-based attention of participants to a specific color, the spatiotemporal characteristics of eye fixations for DOF and FG might be modulated. In our analyses, the discussions rely on the comparison between the DOF and FG tasks in which the similar patches with identical color combination but a slight spatial translation were presented. The effects of color might be canceled out in the present discussions because various types of mirror images are used in our experiment (see section “Materials and Methods”). Studies on the interaction between colors and FG segregation are expected for further understanding.

Our present experiment did not show the significant dependence of perceptual responses and eye movements on the dimension of the cue. A plausible explanation for the cue dimension independence is the effects of the mask screen. In our experiment, a mask was given for 500 ms between the cue and the test stimulus (**Figure 2**). If the dimension of the cue were in fact important, it should have been eliminated by the mask which was identical throughout all trials.

We analyzed the DFF under the FG segregation and the DOF discrimination tasks to examine the mechanism underlying the perception of the figural region. One might argue that the second and subsequent eye fixations during the presentation of stimuli, specifically between 100 and 1000 ms after the onset, provide further evidence for understanding the mechanism of the FG segregation. Interestingly, we observed faster velocities of eye fixations in the FG task compared to that in the DOF task during 0–100 ms while no significant difference after 100 ms (**Supplementary Figure S1**). However, due to slightly sparse sampling rate of the eyetracker machine, we consider that the present estimated velocity does not have enough precision for further analyses and discussions. It would be interesting to examine the second and subsequent eye fixations that could provide further evidence for understanding the mechanism and strategy for the perception of figural regions.

As described previously, a variety of computational models have been proposed for the neuronal mechanism underlying the BO determination and FG segregation. Craft et al. (2007) and Mihalas et al. (2011) proposed the grouping-cell hypothesis for the neural mechanisms underlying the BO selectivity. The grouping-cell mechanism based on the integration of the responses of model BO-selective cells (Russell et al., 2014) indicated markedly greater prediction accuracy on human gaze than the saliency map model based on primary visual features in early-level visual areas (Itti et al., 1998; Itti and Koch, 2001). Grant et al. (2017) implied that biologically plausible learning rules based on modulatory feedback underlie the establishment of the neural network for BO selectivity. Whereas these models gave important predictions for the mechanism of BO coding, details of the FG segregation, and organization have not been clarified. The grouping cells generated a representation of the visual scene in terms of proto-objects and provided rough shapes of objects in the scene (Russell et al., 2014), which did not correspond to the figural region. Poort et al. (2012) developed the network model for understanding the mechanism of FG modulation observed by their physiological experiments. This model appeared to qualitatively reproduce the figure enhancement and ground suppression in V1 and V4. However, this network was rather abstract in that the model did not consider the BO assignment along the stimulus border. Further computational studies are necessary for understanding the neural mechanisms underlying our psychophysical results.

The representation of object regions by BO may be versatile in complex scenes such as natural images where self-occlusion and mutual occlusion often take place (Williford and von der Heydt, 2013). For instance, in a real image of the monkey, one hand may occlude its body while the region of the hand continues to the body through the shoulder. It is difficult to define a two-dimensional (2D) surface of the animal without throwing away some contours. This is natural because three-dimensional (3D) structure is projected onto a 2D surface. Recent physiological studies reported that the cortical representation of 3D objects is mediated by 3D surface and 3D medial axis in monkey visual area IT (Yamane et al., 2008; Hung et al., 2012). It might be interesting if those 3D representations were generated directly from the local BO signals (Hu et al., 2016). This might be in line with the point that occlusion seems to be the most effective cue in the determination of the order of 3D depth. On the other hand, the extraction of the object region from the rest of retinal images might be more important than the representation of the detailed 3D structure in certain cases. BO signals are, in essence, independent of the concept of an object. The representation of the region where an object occupies, either a 2D surface or a 3D space, seems to be a crucial step in between the representation of BO and 3D object. Such intermediate-level representation appears to be necessary and effective in the construction of the representation of 3D objects. Investigations on the link between local BO and global FG are expected to help with understanding the transition of the cortical representation from the pixel-based retinal images to the reconstruction of the 3D world.

## Author Contributions

KS and MU organized the psychophysical experiments. MU developed programs for experiments and performed psychophysical experiments. NW and MU analyzed data of experiments. NW and KS wrote the manuscript for this work.

## Conflict of Interest Statement

The authors declare that the research was conducted in the absence of any commercial or financial relationships that could be construed as a potential conflict of interest.
