# Supplementary material for: Interactions Elicited by the Contradiction Between Figure Direction Discrimination and Figure-Ground Segregation
Source: Front Psychol. 2018 Sep 6;9:1681. doi: 10.3389/fpsyg.2018.01681 (PMC6135913; doi:10.3389/fpsyg.2018.01681)
Supplement: Supplementary file 1 [file Data_Sheet_1.PDF]

Supplementary Material

Interactions elicited by the contradiction between figure direction discrimination and figure-ground segregation

Nobuhiko Wagatsuma*, Mika Uraba, Ko Sakai*

*** Correspondence:** Nobuhiko Wagatsuma: nwagatsuma@is.sci.toho-u.ac.jp,

Ko Sakai: sakai@cs.tsukuba.ac.jp

1 Preliminary experiments for the perception of transparency

Since the perception of transparency depended on individuals (Beck et al., 1984), the α value (see eqs. (1)-(4) in the main text) for each participant were assigned to generate the most transparent surfaces, which were determined based on the results of the preliminary experiments. For the preliminary experiments, the α_{front} and α_{back} were selected from three levels (α = 0.3, 0.5 or 0.7). The combination of three transparency levels yielded 9 patterns for the pair of α_{front} and α_{back}. We used two types of contradictory stimulus that were not included in the stimulus sets for our main experiments (Figure 1(b)). In total, we prepared 144 stimuli (2 types of shape × 2 mirror images × 2 color patterns × 2 patterns for the translation × 9 patterns for the pair of α_{front} and α_{back}). The combination of these stimuli and five repeats produced a total of 720 trials.

The preliminary experiments started with the presentation of a mask display at the screen center for 2000 ms. After the disappearance of the mask, a stimulus (9° × 9°) was presented at the center for 1000 ms. At the end of each trial, a blank screen was given until the detection of participants' perceptual responses. Participants rated the presented stimuli with respect to the degree of transparency of a surface by using keyboards. This total score for the transparency degree was utilized as the index for the perception of transparency.

Table S1 represented the pair of α_{front} and α_{back} for inducing great degree of the transparency for each participant. We regulated contradictory stimuli for our main experiment by using these α values, which induced the interaction between the local DOF discrimination and the global FG segregation.

2 Supplementary Figure

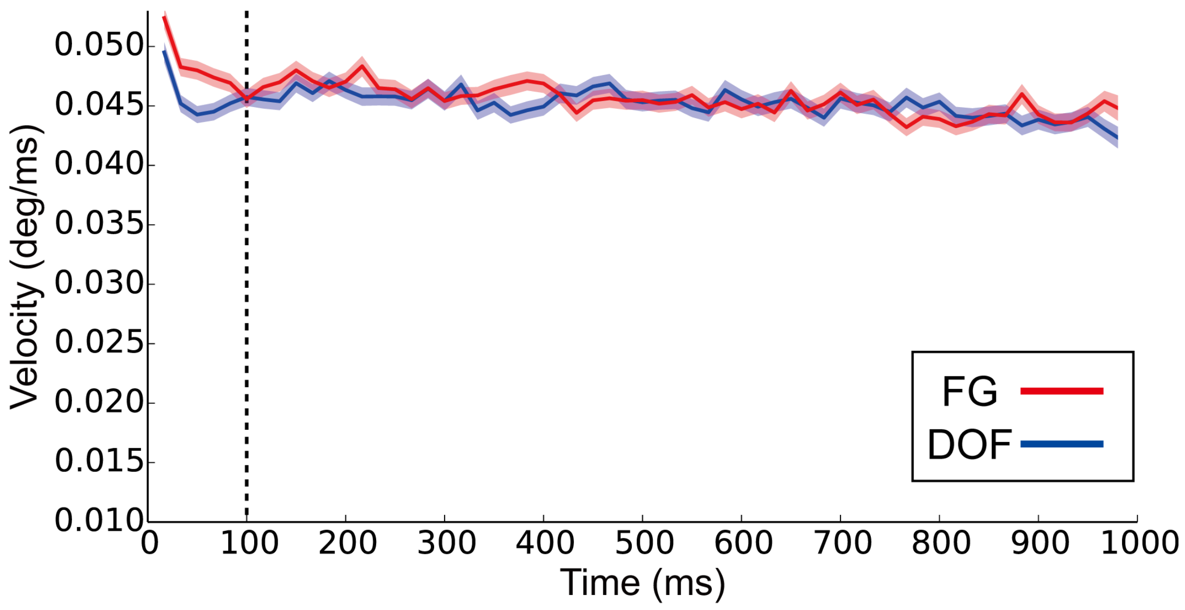

Figure S1. The velocity of the eye movements for the local DOF discrimination and global FG segregation during the presentation of the stimuli (Test). Red and blue lines present the mean velocities under the FG segregation and DOF discrimination task. Shading of curves indicates the standard error among six participants. We observed significantly faster velocity in the FG segregation task than that in the DOF discrimination task for the early time period of 0-100ms. In contrast, there was no significant difference in velocity between two tasks during 100-1000ms.

3 Supplementary Table

Table S1. Results of preliminary experiments. Under these α values, participants markedly perceived transparent surfaces.

	α **values**	
**Participant**	**Red surface**	**Green surface**
A	0.3	0.5
B	0.3	0.3
C	0.7	0.7
D	0.3	0.3
E	0.5	0.3
F	0.7	0.3

References

Beck, J., Prazdny, K., & Ivry, R. (1984). The perception of transparency with achromatic colors, *Perception and Psychophysics*, 35, 407-422, doi: https://doi.org/10.3758/BF03203917.
